# Prospective study on gynaecological effects of two antioestrogens tamoxifen and toremifene in postmenopausal women

**DOI:** 10.1054/bjoc.2001.1703

**Published:** 2001-04

**Authors:** M B Marttunen, B Cacciatore, P Hietanen, S Pyrhönen, A Tiitinen, T Wahlström, O Ylikorkala

**Affiliations:** Departments of Obstetrics and Gynecology, Helsinki University Central Hospital, P.O. Box 140, FIN-00029 HYKS, Finland; Department of Oncology, Helsinki University Central Hospital, P.O. Box 180, FIN-00029 HYKS, Finland; Department of Pathology, Helsinki University Central Hospital, P.O. Box 140, FIN-00029 HYKS, Finland

**Keywords:** breast cancer, antioestrogens, tamoxifen, toremifene, endometrium

## Abstract

To assess and compare the gynaecological consequences of the use of 2 antioestrogens we examined 167 postmenopausal breast cancer patients before and during the use of either tamoxifen (20 mg/day, *n* = 84) or toremifene (40 mg/day, *n* = 83) as an adjuvant treatment of stage II–III breast cancer. Detailed interview concerning menopausal symptoms, pelvic examination including transvaginal sonography (TVS) and collection of endometrial sample were performed at baseline and at 6, 12, 24 and 36 months of treatment. In a subgroup of 30 women (15 using tamoxifen and 15 toremifene) pulsatility index (PI) in an uterine artery was measured before and at 6 and 12 months of treatment. The mean (±SD) follow-up time was 2.3 ± 0.8 years. 35% of the patients complained of vasomotor symptoms before the start of the trial. This rate increased to 60.0% during the first year of the trial, being similar among patients using tamoxifen (57.1%) and toremifene (62.7%). Vaginal dryness, which was present in 6.0% at baseline, increased during the use of tamoxifen (26.2%) and toremifene (24.1%). Endometrial thickness increased from baseline (3.9 ± 2.7 mm) to 6.8 ± 4.2 mm at 6 months (*P*< 0.001), and no difference emerged between the 2 regimens in this regard. Before the start of the antioestrogen regimen, the endometrium was atrophic in 71 (75.5%) and proliferative in 19 of 94 (20.2%) samples; 4 patients had benign endometrial polyps. During the use of antioestrogen altogether 339 endometrial samples were taken (159 in tamoxifen group, 180 in toremifene group). The endometrium was proliferative more often in the tamoxifen group (47.8%) than in the toremifene group (32.2%) (*P*< 0.0001). 20 patients had a total of 24 polyps (17 in tamoxifen and 9 in toremifene group, *P*< 0.05) during the use of antioestrogens. One patient in the toremifene group developed endometrial adenocarcinoma at 12 months, and one patient had breast cancer metastasis on the endometrium. Tamoxifen failed to affect the PI in the uterine artery, but toremifene reduced it by 15.0% (*P*< 0.05) by 12 months. In conclusion, tamoxifen and toremifene cause similarly vasomotor and vaginal symptoms. Neither regimen led to the development of premalignant endometrial changes. Our data suggest that so close endometrial surveillance as used in our study may not be mandatory during the first 3 years of use of antioestrogen treatment. © 2001 Cancer Research Campaign http://www.bjcancer.com


British Journal of Cancer (2001) 84(7), 897–902
© 2001 Cancer Research Campaign
doi: 10.1054/ bjoc.2001.1703, available online at http://www.idealibrary.com on IDEAL®

### Prospective study on gynaecological effects of two antioestrogens tamoxifen and toremifene in postmenopausal women

**MB Marttunen[1], B Cacciatore[1], P Hietanen[2], S Pyrhönen[2], A Tiitinen[1], T Wahlström[3] and O Ylikorkala[1]**

[1]Departments of Obstetrics and Gynecology, Helsinki University Central Hospital, P.O. Box 140, FIN-00029 HYKS, Finland; [2]Department of Oncology, Helsinki University Central Hospital, P.O. Box 180, FIN-00029 HYKS, Finland; [3]Department of Pathology, Helsinki University Central Hospital, P.O. Box 140, FIN-00029 HYKS, Finland

**Summary** To assess and compare the gynaecological consequences of the use of 2 antioestrogens we examined 167 postmenopausal breast cancer patients before and during the use of either tamoxifen (20 mg/day, $n = 84$) or toremifene (40 mg/day, $n = 83$) as an adjuvant treatment of stage II–III breast cancer. Detailed interview concerning menopausal symptoms, pelvic examination including transvaginal sonography (TVS) and collection of endometrial sample were performed at baseline and at 6, 12, 24 and 36 months of treatment. In a subgroup of 30 women (15 using tamoxifen and 15 toremifene) pulsatility index (PI) in an uterine artery was measured before and at 6 and 12 months of treatment. The mean (±SD) follow-up time was 2.3 ± 0.8 years. 35% of the patients complained of vasomotor symptoms before the start of the trial. This rate increased to 60.0% during the first year of the trial, being similar among patients using tamoxifen (57.1%) and toremifene (62.7%). Vaginal dryness, which was present in 6.0% at baseline, increased during the use of tamoxifen (26.2%) and toremifene (24.1%). Endometrial thickness increased from baseline (3.9 ± 2.7 mm) to 6.8 ± 4.2 mm at 6 months ($P < 0.001$), and no difference emerged between the 2 regimens in this regard. Before the start of the antioestrogen regimen, the endometrium was atrophic in 71 (75.5%) and proliferative in 19 of 94 (20.2%) samples; 4 patients had benign endometrial polyps. During the use of antioestrogen altogether 339 endometrial samples were taken (159 in tamoxifen group, 180 in toremifene group). The endometrium was proliferative more often in the tamoxifen group (47.8%) than in the toremifene group (32.2%) ($P < 0.0001$). 20 patients had a total of 24 polyps (17 in tamoxifen and 9 in toremifene group, $P < 0.05$) during the use of antioestrogens. One patient in the toremifene group developed endometrial adenocarcinoma at 12 months, and one patient had breast cancer metastasis on the endometrium. Tamoxifen failed to affect the PI in the uterine artery, but toremifene reduced it by 15.0% ($P < 0.05$) by 12 months. In conclusion, tamoxifen and toremifene cause similarly vasomotor and vaginal symptoms. Neither regimen led to the development of premalignant endometrial changes. Our data suggest that so close endometrial surveillance as used in our study may not be mandatory during the first 3 years of use of antioestrogen treatment. © 2001 Cancer Research Campaign http://www.bjcancer.com

**Keywords**: breast cancer; antioestrogens; tamoxifen; toremifene; endometrium

Antioestrogenic tamoxifen is widely used for the treatment of breast cancer (Early Breast Cancer Trialists Collaborative Group, 1998) and even for the prevention of breast cancer in high-risk patients (Fisher et al, 1998). Toremifene is another antioestrogen, which has been proven to be effective in the treatment of breast cancer (Hayes et al, 1995; Wiseman and Goa, 1997). Because breast cancer is the most common cancer of women (Garfinkel et al, 1994), antioestrogens have become one of the commonest types of drugs used by women.

Antioestrogens act by blocking oestrogen receptors not only in breast cancer cells but also in reproductive organs possessing these receptors. Therefore, use of antioestrogens can cause a number of gynaecological consequences (Cohen et al, 1994; Barakat, 1996). It is known, mostly from cross-sectional studies, that antioestrogen use is associated with vasomotor symptoms and vaginal dryness and irritation (Buckley and Goa, 1989; Wiseman and Goa, 1997), which may be signs and consequences of hypooestrogenism. In the uterus, and especially in the endometrium, the effect of antioestrogens resembles that of oestrogens (Lahti et al, 1993; Friedl and Jordan, 1994), and in this regard tamoxifen may be more potent than toremifene at least in animal experiments (DiSalle et al, 1990). The use of both tamoxifen and toremifene has been linked to the risk of endometrial polyps (Tomás et al, 1995; Barakat, 1996) and tamoxifen may increase the risk of endometrial cancer (Fisher et al, 1994; Rutqvist et al, 1995; Fisher et al, 1998; Early Breast Cancer Trialists Collaborative Group, 1998). However, these data have been collected mostly from cross-sectional studies, which are prone to several methodological weaknesses. Therefore we designed a prospective comparative trial to assess gynaecological consequences of the use of tamoxifen and toremifene in patients with breast cancer.

Received 3 April 2000
Revised 19 December 2000
Accepted 19 January 2001

Correspondence to: M Marttunen

### PATIENTS AND METHODS

With the permission of the local ethics committee, we studied 167 postmenopausal (more than 6 months since their last menstrual period, FSH > 40 IU/l) patients with stage II–III breast cancer (Table 1). The patients had undergone surgery for breast cancer 6–8

**Table 1** Clinical characteristics of the study population ($n$ = 167) using either tamoxifen (20 mg/day, $n$ = 84), or toremifene (40 mg/day, $n$ = 83) (mean ± SD), (range) or (percent)

| | Tamoxifen ($n$ = 84) | Toremifene ($n$ = 83) | All ($n$ = 167) |
|---|---|---|---|
| Age (y) | 63.2 ± 7.8 (48–77) | 62.4 ± 8.9 (44–81) | 62.8 ± 8.4 (44–81) |
| Age at | | | |
| Menarche | 13.3 ± 1.6 (9–18) | 13.7 ± 1.9 (9–19) | 13.5 ± 1.8 (9–19) |
| Menopause | 49.9 ± 3.5 (40–58) | 48.8 ± 4.2 (33–56) | 49.4 ± 3.9 (33–58) |
| Nulliparous | 29 (34.5%) | 16 (19.3%) | 45 (26.9%) |
| Body mass index (kg/m$^2$) | 27.4 ± 0.5 (19–40) | 27.0 ± 0.5 (19–38) | 27.2 ± 0.3 (19–40) |
| Hysterectomized | 21 (25.0%) | 17 (20.5%) | 38 (22.7%) |
| Previous use of HRT | 37 (44.0%) | 36 (42.9%) | 73 (43.7%) |
| Use of antihypertensive drugs | 29 (34.5%) | 24 (28.9%) | 53 (31.7%) |
| Smoking | 8 (9.5%) | 19 (22.9%) | 27 (16.2%) |
| Breast cancer classification | | | |
| Tumour size | | | |
| T1 | 49 (58.3%) | 38 (45.8%) | 87 (52.1%) |
| T2 | 30 (35.7%) | 39 (47.0%) | 69 (41.3%) |
| T3 | 5 (6.0%) | 6 (7.2%) | 11 (5.6%) |
| Nodal status | | | |
| N1 | 83 (98.8%) | 80 (96.4%) | 163 (97.6%) |
| N2 | 1 (1.2%) | 3 (3.6%) | 4 (2.4%) |
| Surgery | | | |
| Radical | 55 (65.5%) | 56 (67.5%) | 111 (66.5%) |
| Conservative | 29 (34.5%) | 27 (32.5%) | 56 (33.5%) |

weeks before entering this study, which is a part of the Finnish Breast Cancer Group Adjuvant Toremifene versus Tamoxifen Trial (FBCG II) (Holli, 1998). The cancer had spread to the axillary nodes in all patients, but no other metastases were detected in a thorough clinical examination. The patients received local radiotherapy 5 times a week for 5 weeks, and were randomized to start a 3-year course either with tamoxifen (20 mg/day, Tadex®, Orion, Turku, Finland), or with toremifene (40 mg/day, Fareston®, Orion, Turku, Finland), which are expected to have similar efficacy in the prevention of cancer recurrence (Hayes et al, 1995; Wiseman and Goa, 1997; Pyrhönen et al, 1999). The study groups did not differ from each other with regard to relevant clinical data, such as age, history of hysterectomy, or the use of oestrogen replacement therapy before the diagnosis of breast cancer (Table 1). The patients were otherwise healthy, but 53 patients (29 in tamoxifen group, 24 in toremifene group) used different antihypertensive drugs (beta-blockers, diuretics, calcium channel blockers) to control elevated blood pressure, and 27 women smoked (8 in tamoxifen and 19 in toremifene group).

Before the start of antioestrogen, all patients underwent a careful clinical examination (weight, height, blood pressure), and pelvic examination was also carried out. Pap smears were collected, and transvaginal sonographic (TVS) examination (Aloka SSD 500) was performed in all patients with a uterus and/or ovaries to assess pelvic anatomy, including endometrial thickness. If endometrial thickness (double layer) exceeded 8 mm or if an endometrial polyp was suspected, 10–20 ml of sterile saline were injected into the uterine cavity and TVS was repeated (Widrich et al, 1996). Any polyp was removed through hysteroscopy and examined under microscope. Endometrial biopsies were collected (Pipelle endometrial aspirator, Unimar, Wilmington, CN) (Chambers and Chambers, 1992) and examined under microscope by the same experienced pathologist (TW). The histology of the endometrium was classified as atrophic (small simple, tubular glands lined by cuboidal epithelium, the nuclei are centrally placed, no mitoses seen) or mildly proliferative (small tubular glands lined by cuboidal to columnar epithelium, the nuclei are ovoid and basally or centrally located, some mitoses are seen). The patients were seen at 6 months, 12 months, 24 months and 36 months, and at each visit the examinations done at recruitment were repeated. In addition, at recruitment and at each follow-up visit the patients were carefully interviewed for gynaecological symptoms, possible adverse effects, and for the occurrence of uterine bleeding. Patients experiencing uterine bleeding at any time during the follow-up were instructed to report at the research centre immediately, and TVS and endometrial biopsy were repeated.

To study the effect of antioestrogens on pulsatility index (PI) of the uterine artery, we selected a subgroup of 30 patients (15 using tamoxifen, 15 using toremifene), who were studied with Doppler equipment (Hitachi 515A, Tokyo, Japan, 6.5 MHz endovaginal transducer) before the start of medication and at 6 and 12 months of trial, as described before (Cacciatore et al, 1998). These assessments were always done by the same investigator (BC), who was blinded to the code of the treatment.

The numerical variables of the groups were compared with Student's two-tailed unpaired $t$-test. The changes in variables within the groups were first subjected to the analysis of variance and if this showed a difference, the significance of the difference was tested by paired $t$-test. The comparison of two proportions in case of paired samples (e.g. endometrial samples) was performed by McNemar's test. Pearson Chi-square statistics were applied when comparing the distribution of categorical variables of 2 independent samples. The data are presented as mean ± SD. A $P$ value of <0.05 was considered significant.

### RESULTS

A total of 167 patients have been followed for a mean of 2.3 ± 0.8 years. All patients completed one year follow-up, 130 patients (61 tamoxifen users, 69 toremifene users) 2 years follow-up, and 94 patients (42 tamoxifen users, 52 toremifene users) 3 years follow-up. The trial is still going on in 36 patients. 17 patients (11 in tamoxifen group, 6 in toremifene group) failed to report according to the protocol, and 20 patients discontinued the use of antioestrogens, 5 because of adverse effects (3 in tamoxifen group, 2 in toremifene group) and 15 because of recurrence of breast cancer (7 in tamoxifen group, 8 in toremifene group). Thus 37 patients have discontinued the follow-up.

Vasomotor symptoms, which were complained by 59 patients (35.3%) (32 in tamoxifen, 27 in toremifene group) before the trial, increased in frequency but were equally common and severe in both groups (Table 2). Vaginal symptoms (dryness, irritation, discharge) increased during the use of antiestrogen in both groups (Table 2).

Small uterine fibroids (<4 cm) were detected in 13 patients starting tamoxifen and in 15 patients starting toremifene. Neither tamoxifen nor toremifene affected the size of the fibroids or caused new fibroids. Simple ovarian cysts (<3 cm) were detected in 4 women starting toremifene, but they disappeared during the first 6 months on toremifene. Thecoma on the left ovary

© 2001 Cancer Research Campaign

**Table 2** Vasomotor and vaginal symptoms before and during first year's tamoxifen (20 mg day$^{-1}$, $n$ = 84) or toremifene (40 mg day$^{-1}$, $n$ = 83) use in postmenopausal patients with breast cancer

| | Tamoxifen | Toremifene |
|---|---|---|
| **Vasomotor symptoms** | | |
| Before | 32 (38.1%) | 27 (32.5%) |
| Mild | 12 (14.3%) | 16 (19.8%) |
| Moderate | 10 (11.9%) | 11 (13.3%) |
| Severe | 1 (1.2%) | 0 |
| During trial | 48 (57.1%) | 52 (62.7%) |
| Mild | 18 (21.4%) | 13 (15.7%) |
| Moderate | 16 (19.0%) | 27 (32.5%) |
| Severe | 14 (21.4%) | 12 (14.5%) |
| **Vaginal irritation or dryness** | | |
| Before | | |
| Dryness | 5 (5.9%) | 5 (6.0%) |
| During trial | | |
| Dryness | 8 (9.5%) | 5 (6.0%) |
| Irritation | 8 (9.5%) | 4 (4.8%) |
| Discharge | 6 (7.1%) | 11 (13.2%) |

(50 × 45 × 62 mm) was diagnosed in one patient after one year's use on tamoxifen; it was removed laparoscopically. Before the start of antioestrogen, 5 women (3 in tamoxifen group, 2 in toremifene group) complained of symptoms of a partial prolapse of the uterus. The antioestrogen regimen failed to affect these symptoms, and vaginal hysterectomy was performed in these patients during the trial; the symptoms were relieved. Pap smears revealed no pre-malignant lesions before or during the trial in any patient.

The mean endometrial thickness, which was 3.9 ± 2.7 mm before the start of antioestrogen, increased to 6.8 ± 4.2 mm ($P$ < 0.001) already during the first 6 months but no further thickening was seen later (Table 3). There were no differences between the tamoxifen and toremifene groups in this regard. Age or time since the onset of menopause did not correlate with the increase in endometrial thickness (data not shown). In total, vaginal bleedings occurred in 13 women (10.1%) during the trial (5 in tamoxifen group, 8 in toremifene group) between 6 months and 2.9 years of use of antioestrogens. Of these patients, endometrial polyps were detected in 4 and endometrium showed mild proliferation in 8 and atrophy in one patient.

Tamoxifen failed to affect PI in the uterine artery at 6 or 12 months, but toremifene reduced it from 2.24 ± 0.65 at baseline to 1.85 ± 0.69 at 12 months ($P$ < 0.05). The changes in PI or endometrial thickness did not correlate with each other in the whole series or in either subgroup.

Before the initiation of antioestrogens, adequate endometrial samples were obtained from 94 of 129 patients (73%) with intact uterus (44 in tamoxifen group, 50 in toremifene group), whereas endometrial sampling did not succeed in 35 patients due to cervical stenosis ($n$ = 25) or intolerable pain ($n$ = 10) (Table 3). At baseline the endometrium was atrophic in 71 patients (75.5%) and proliferative in 19 patients (20.2%) and contained polyps in 4 women (Table 3). Of the 19 patients with proliferative endometrium, 16 had used oestrogen replacement therapy until the diagnosis of breast cancer. Tamoxifen increased the mean incidence of proliferative endometrium more (from 20.4% to 46.8%) than toremifene (from 20.0% to 32.2%) did ($P$ < 0.0001) (Table 3). Endometrial histology was not relative to endometrial thickness in the whole series or in either of the subgroups (Figure 1). The diagnosis of endometrial polyp was made 24 times (17 in tamoxifen group, 7 in toremifene group, $P$ < 0.05), in 20 patients, during the study period (Table 3). All these polyps were removed hysteroscopically. Polyps did not recur in patients whose polyps were removed at entry. 3 patients using tamoxifen and one patient using toremifene developed polyps repeatedly at 1 or 2 years intervals during the trial.

One patient using toremifene proved to have endometrial adenocarcinoma at 12 months. She had no bleeding but the endometrium was thick (12 mm) and contained a polyp. The polyp, which was removed through hysteroscopy, showed hyperplasia, and hysterectomy with bilateral oophorectomy was performed. Well-differentiated adenocarcinoma limited to the endometrium was detected in a histological examination. In addition, both ovaries contained small metastases of breast cancer. In another woman using tamoxifen and reporting no bleeding, endometrial thickness was 7 mm at 12

**Table 3** Endometrial findings in postmenopausal breast cancer patients starting the use of either tamoxifen (20 mg/day, $n$ = 63) or toremifene (40 mg/day, $n$ = 66)

| | Before | At 6 months | *P* value* | 12 months | *P* value* | 24 months | *P* value* | 36 months | *P* value* |
|---|---|---|---|---|---|---|---|---|---|
| Tamoxifen | | | | | | | | | |
| biopsy succeeded | 44/63 (69.8%) | 48/63(76.2%) | | 50/63 (79.4%) | | 35/45 (77.8%) | | 26/29 (89.7%) | |
| atrophy | 34 (77.3%) | 18 (37.5%) | <0.001 | 23 (46.0%) | <0.005 | 14 (40.0%) | <0.005 | 10 (38.5%) | <0.05 |
| mild proliferation | 9 (20.4%) | 25 (52.1%) | <0.001 | 23 (46.0%) | <0.05 | 16 (45.7%) | <0.05 | 12 (46.1%) | NS |
| polyp | 1 (2.3%) | 5 (10.6%) | NS | 4 (8.0%) | NS | 4 (11.4%) | <0.05 | 4 (15.4%) | <0.05 |
| carcinoma | 0 | 0 | | 0 | | 1[#] (2.9%) | | 0 | |
| endometrial thickness (mm), (mean ± SD) | 3.5 ± 1.9 | 7.5 ± 4.8 | <0.001 | 7.1 ± 3.5 | <0.001 | 7.8 ± 4.5 | <0.001 | 7.6 ± 3.9 | <0.001 |
| Toremifene | | | | | | | | | |
| biopsy succeeded | 50/66 (75.8%) | 50/66 (75.8%) | | 55/66 (83.3%) | | 46/52 (88.5%) | | 29/35 (82.9%) | |
| atrophy | 37 (74.0%) | 37 (74.0%) | NS | 29 (52.7%) | <0.05 | 28 (60.9%) | NS | 20 (69.0%) | NS |
| mild proliferation | 10 (20.0%) | 11 (22.0%) | NS | 25 (45.5%) | <0.005 | 15 (32.6%) | NS | 7 (24.1%) | NS |
| polyp | 3 (6.0%) | 2 (4.0%) | NS | 0 | NS | 3 (6.5%) | NS | 2 (6.9%) | NS |
| carcinoma | 0 | 0 | | 1[§] (1.8%) | | 0 | | 0 | |
| endometrial thickness (mm), (mean ± SD) | 4.2 ± 3.2 | 6.2 ± 3.5 | <0.001 | 7.1 ± 4.0 | <0.001 | 7.3 ± 3.1 | <0.001 | 7.7 ± 4.3 | <0.001 |

* Compared with initial; [#] breast cancer metastasis on endometrium; [§] endometrial adenocarcinoma gr 1.

© 2001 Cancer Research Campaign

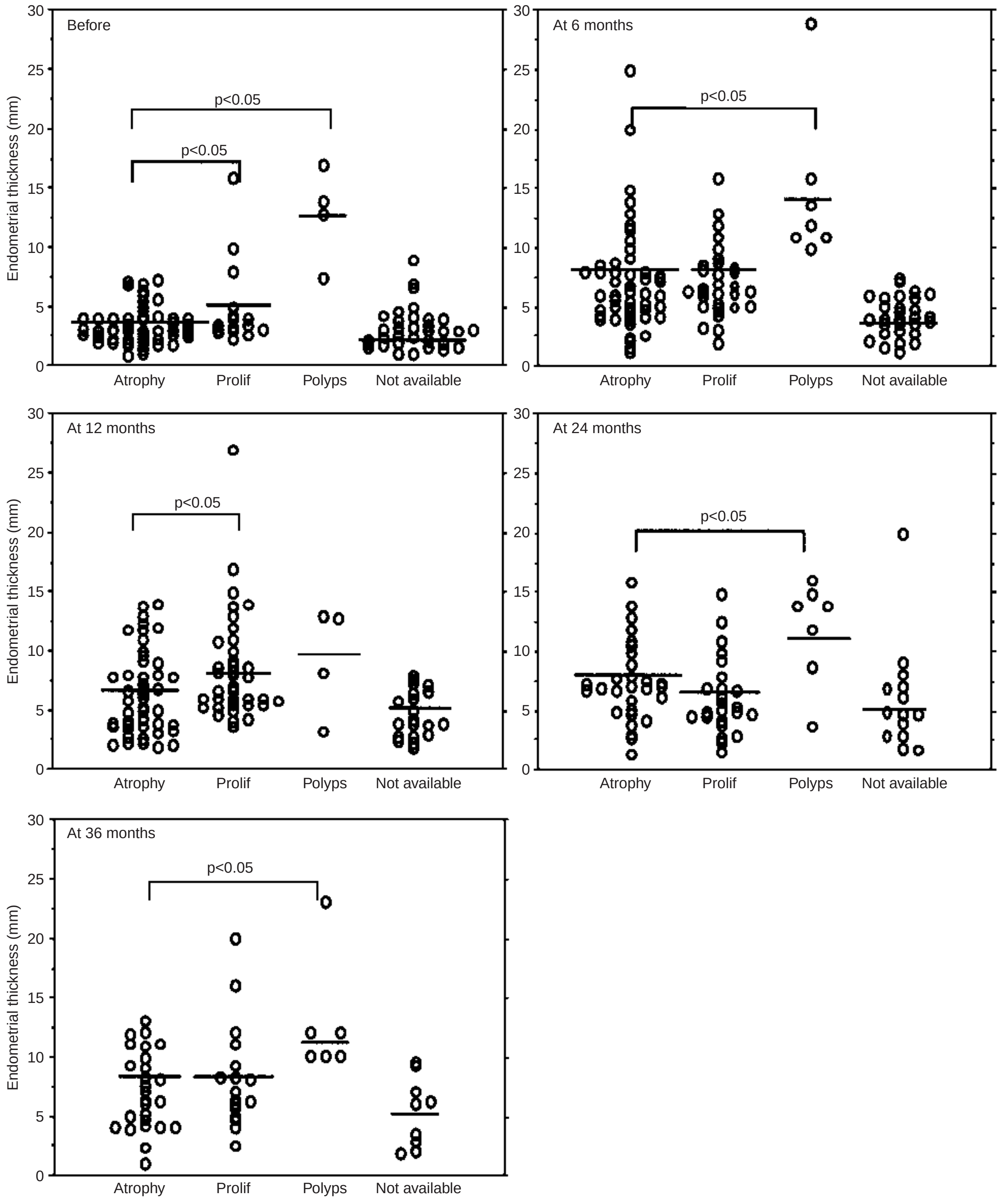

**Figure 1** Endometrial thickness compared to endometrial histology in 129 postmenopausal patients using either tamoxifen ($n = 63$) or toremifene ($n = 66$)

months, and a biopsy revealed metastasis of breast cancer. At laparotomy, metastases of breast cancer were seen in all pelvic and intra-abdominal organs.

### DISCUSSION

The use of antioestrogens has drastically increased in modern clinical practice (Garfinkel et al, 1994; Fisher et al, 1998). Therefore it is important to assess all the possible consequences of the use of antioestrogens, appearing also outside of the breasts, and our study focused on gynaecological findings and symptoms. It is clear from our study that both tamoxifen and toremifene caused and/or worsened vasomotor symptoms, although these regimens did not differ from each other in this regard. In previous cross-sectional studies, the occurrence of severe hot flushes during antioestrogen regimens has varied between 20 and 30% (Buckley and Goa, 1989; Love

© 2001 Cancer Research Campaign et al, 1991; Hayes et al, 1995). Compared with this rate the frequency of hot flushes was higher in our study (60%), but 33–38% of all study patients reported these symptoms already at baseline examination. Vaginal dryness and irritation were also common prior to the trial but they increased during both regimens; this is in agreement with a previous cross-sectional study (Hayes et al, 1995). Thus our data imply that tamoxifen and toremifene probably cause a hypo-oestrogenic milieu at least in the vagina, because vaginal dryness was a common complaint. Such a hypo-oestrogenic effect was not seen in the endometrium. It is difficult to deduce why the vagina and endometrium behave so differently in regard to antioestrogens, but it is probably related to different receptors status in these organs. However, only 5 patients (3%) discontinued the use of antioestrogens because of hypo-oestrogenic adverse effects.

The use of tamoxifen and toremifene led to a similar thickening of the endometrium already at 6 months follow-up and this effect was maintained during the whole trial. This finding is in accordance with one previous prospective study (Tomás et al, 1995). The mechanism of endometrial thickening is not fully understood, but it could be a reflection of the oestrogenic effect of antioestrogens. This is supported by the appearance of a proliferative endometrium, although no relation between endometrial histology and endometrial thickness was seen. With regard to endometrial effect, tamoxifen and toremifene appear fairly similar, although the incidence of proliferative endometrium was higher in tamoxifen users. Furthermore, the appearance of endometrial polyps may be a reflection of oestrogenic effect. However, what is noteworthy in our data is that endometrial thickening in antioestrogen users is not a sign of endometrial proliferation but in effect, a thick endometrium can be atrophic. This has been detected also in some previous studies (Cohen et al, 1993; Touraine et al, 1995; Ismail, 1996). Thus, endometrial thickening is probably a result of the enlargement of subendometrial glands (Goldstein, 1994; Neven, 1995; McGonigle et al, 1998) and is no indication for endometrial biopsy.

Toremifene reduced significantly the PI in the uterine artery by 12 months whereas tamoxifen, contrary to the results of a previous study (Kedar et al, 1994), had no effect on PI. We did not have a control group, but in view of previous data (Hillard et al, 1992; Pirhonen et al, 1993) it is unlikely that one year's increase in age would have reduced the PI; in contrast, it would rather have increased it. Pulsatility index reflects a resistance to blood flow distal to the site of measurement and, thus, its reduction is a sign of decreased vascular resistance. Consequently, toremifene may have caused a true vasodilatation in the uterine artery and, in this regard, the effect of toremifene resembles that of oestrogens (Cacciatore et al, 1998). The lacking effect of tamoxifen on Pl may suggest a weaker oestrogenic effect on uterine vasculature but, of course, the rather small number of patients limits any firm conclusions. It was also of interest that the changes in endometrial thickness and in PI were in no relation to each other. This may suggest that antioestrogens operate through different mechanisms of action on the endometrium and on vascular wall.

Endometrial safety during long-term use of antioestrogens has caused much concern among gynaecologists. This issue is important, since a history of breast cancer, alone, denotes a 1.6-fold risk of endometrial cancer (Adami et al, 1997). The risk of endometrial carcinoma in tamoxifen users has been shown to be increased in some (Fisher et al, 1994; Rutqvist et al, 1995; Fisher et al, 1998) although not all studies (Ribeiro and Swindell, 1988; Stewart et al, 1996), and close endometrial surveillance has been recommended to these patients (Fornander et al, 1993; van Leeuwen et al, 1994; Tesoro et al, 1999). We followed prospectively a rather large number of postmenopausal patients, whose endometrial status was carefully examined before and during the trial. Both regimens, used for a mean of 2.3 years, were associated with endometrial thickening and with an increased incidence of proliferative endometrium. However, only one patient showed a hyperplastic polyp and primary endometrial carcinoma. Thus the risk of endometrial carcinoma in the whole study population (0.03/100 woman-years) is even smaller than in a Finnish normal population in this age group (0.06/100 woman-years; Finnish Cancer Register, 1995). Thus our data do not lend any support to the fear that antioestrogens increase the risk of endometrial carcinoma at least in the first 2–3 years of use. Our data seriously question the rationale of endometrial surveillance of antioestrogen users (Fornander et al, 1993; Tesoro et al, 1994; van Leeuwen et al, 1994).

### REFERENCES

Adami H-O, Bergström R, Weiderpass E, Persson I, Barlow L and McLaughlin JK (1997) Risk for endometrial cancer following breast cancer: a prospective study in Sweden. *Cancer Causes Control* **8**: 821–827

Barakat RR (1996) Tamoxifen and endometrial neoplasia. *Clin Obstet Gynecol* **39**: 629–640

Buckley MMT and Goa KL (1989) Tamoxifen. A reappraisal of its pharmacodynamic and pharmacokinetic properties, and therapeutic use. *Drugs* **37**: 451–490

Cacciatore B, Paakkari I, Toivonen J, Tikkanen MJ and Ylikorkala O (1998) Randomized comparison of oral and transdermal hormone replacement on the carotid and uterine artery resistance to blood flow. *Obstet Gynecol* **92**: 563–568

Chambers JT and Chambers SK (1992) Endometrial sampling: When? Where? Why? With what? *Clin Obstet Gynecol* **35**: 28–39

Cohen I, Rosen DJD, Tepper R, Cordoba M, Altaras MM and Byeth Y (1993) Ultrasonographic evaluation of the endometrium and correlation with endometrial sampling in postmenopausal patients treated with tamoxifen. *J Ultrasound Med* **5**: 275–280

Cohen I, Altaras MM, Lew S, Tepper R, Beyth Y and Ben-Baruch G (1994) Ovarian endometrioid carcinoma and endometriosis developing in a postmenopausal breast cancer patient during tamoxifen therapy: A case report and review of the literature. *Gynecol Oncol* **55**: 443–447

Di Salle E, Zaccheo T and Ornati G (1990) Antiestrogenic and antitumor properties of the new triphenylethylene derivative toremifene in the rat. *J Steroid Biochem* **36**: 203–206

Early Breast Cancer Trialists' Collaborative Group (1998) Tamoxifen for early breast cancer: an overview of the randomised trials. *Lancet* **351**: 1451–1467

Fisher B, Costantino JP, Redmond CK, Fisher ER, Wickerham DL and Cronin WM (1994) Endometrial cancer in tamoxifen-treated breast cancer patients: Findings from the National Surgical Adjuvant Breast and Bowel Project (NSABP) B-14. *J Natl Cancer Inst* **86**: 527–537

Fisher B, Costantino JP, Wickerham DL, Redmond CK, Kavanah M, Cronin WM, Vogel V, Robidoux A, Dimitrov N, Atkins J, Daly M, Wieand S, Tan-Chiu E, Ford L and Wolmark N and other investigators of NSABBP (1998) Tamoxifen for prevention of breast cancer: Report of the National Surgical Adjuvant Breast and Bowel Project P-1 Study. *J Natl Cancer Inst* **90**: 1371–1388

Fornander T, Hellström A-C and Moberger B (1993) Descriptive clinicopathologic study of 17 patients with endometrial cancer during or after adjuvant tamoxifen in early breast cancer. *J Natl Cancer Inst* **85**: 1850–1855

Friedl A and Jordan VG (1994) What do we know and what don't we know about tamoxifen in the human uterus. *Breast Cancer Res Treat* **31**: 27–39

Garfinkel L, Boring CC and Heath Jr. CW (1994) Changing trends. An overview of breast cancer incidence and mortality. *Cancer* **74**: 222–227

Goldstein SR (1994) Unusual ultrasonographic appearance of the uterus in patients receiving tamoxifen. *Am J Obstet Gynecol* **170**: 447–451

Hayes DF, Van Zyl JA, Hacking A, Goedhals L, Bezwoda WR, Mailliard JA, Jones SE, Vogel CL, Berris RF, Shemano I and Schoenfelder J (1995) Randomized comparison of tamoxifen and two separate doses of toremifene in postmenopausal patients with metastatic breast cancer. *J Clin Oncol* **13**: 2556–2566

© 2001 Cancer Research Campaign

Hillard TC, Bourne TH, Whitehead MI, Crayford TB, Collins WP and Campbell S (1992) Differential effects of transdermal estradiol and sequential progestogens on impedance to flow within the uterine arteries of postmenopausal women. *Fertil Steril* **58**: 959–963
Holli K (1998) Adjuvant trials of toremifene vs tamoxifen: The European experience. *Oncology* **12**S: 23–27
Ismail SM (1996) The effects of tamoxifen on the uterus. *Current Opin Obstet Gynecol* **8**: 27–31
Kedar RP, Bourne TH, Powles TJ, Collins WP, Ashley SE, Cosgrove DO and Campbell S (1994) Effects of tamoxifen on uterus and ovaries of postmenopausal women in a randomised breast cancer prevention trial. *Lancet* **343**: 1318–1321
Lahti E, Blanco G, Kauppila A, Apaja-Sarkkinen M, Taskinen PJ and Laatikainen T (1993) Endometrial changes in postmenopausal breast cancer patients receiving tamoxifen. *Obstet Gynecol* **81**: 660–664
Love R, Cameron L, Connell BL and Leventhal H (1991) Symptoms associated with tamoxifen treatment in postmenopausal women. *Arch Intern Med* **151**: 1842–1847
McGonigle KF, Shaw SL, Vasilev SA, Odom-Maryon T, Roy S and Simpson JF (1998) Abnormalities detected on transvaginal ultrasonography in tamoxifen-treated postmenopausal breast cancer patients may represent endometrial cystic atrophy. *Am J Obstet Gynecol* **178**: 1145–1150
Neven P (1995) Endometrial changes in patients on tamoxifen. *Lancet* **346**: 1292
Pirhonen JP, Vuento MH, Mäkinen JI and Salmi TA (1993) Long-term effects of hormone replacement therapy on the uterus and on uterine circulation. *Am J Obstet Gynecol* **168**: 620–623
Pyrhönen S, Ellme'n J, Vuorinen J, Gershanovich M, Tominaga T, Kaufman M and Hayes DF (1999) Meta-analysis of trials comparing toremifene with tamoxifen and factors predicting outcome of antiestrogen therapy in postmenopausal women with breas cancer. *Breast Cancer Res Treat* **56**: 133–143
Ribeiro G and Swindell R (1988) The Christie hospital adjuvant tamoxifen trial: Status at 10 years. *Br J Cancer* **57**: 601–603
Rutqvist LE, Johansson H, Signomklao T, Johansson U, Fornander H and Wilking N (1995) Adjuvant tamoxifen therapy for early stage breast cancer and second primary malignancies. *J Natl Cancer Inst* **87**: 645–651
Stewart HJ, Forrest AP, Everington D, McDonald CC, Dewar JA, Hawkins RA, Prescott RJ and George WD (1996) Randomised comparison of 5 years of adjuvant tamoxifen with continuous therapy for operable breast cancer: the Scottish Cancer Trials Breast Group. *Br J Cancer* **74**: 297–299
Tesoro MR, Borgida AF, Maclaurin NA and Asuncion CM (1999) Transvaginal endometrial sonography in postmenopausal women taking tamoxifen. *Obstet Gynecol* **93**: 363–366
Tomás E, Kauppila A, Blanco G, Apaja-Sarkkinen M and Laatikainen T (1995) Comparison between the effects of tamoxifen and toremifene on the uterus in postmenopausal breast cancer patients. *Gynecol Oncol* **59**: 261–266
Touraine P, Driguez P, Cartier I, Yaneva H, Kuttenn F and Mauvais-Jarvis P (1995) Lack of induction of endometrial hyperplasia with tamoxifen. *Lancet* **345**: 254–255
van Leeuwen FE, Benraadt J, Coebergh JWW, Kiemeney LALM and Gimbre're CHF (1994) Risk of endometrial cancer after tamoxifen treatment of breast cancer. *Lancet* **343**: 448–452
Widrich T, Bradley LD, Mitchinson AR and Collins RI (1996) Comparison of saline infusion sonography with office hysteroscopy for the evaluation of the endometrium. *Am J Obstet Gynecol* **174**: 1327–1334
Wiseman LR and Goa KL (1997) Toremifene. A review of its pharmacological properties and clinical efficacy in the management of advanced breast cancer. *Drugs* **54**: 141–160

 *© 2001 Cancer Research Campaign*
